# Comparative Prognostic Performance of HALP, PIV, and Naples Prognostic Score in Critically Ill Patients with Sepsis: A Retrospective Multicentre Cohort Study

**DOI:** 10.3390/jcm15124729

**Published:** 2026-06-18

**Authors:** Sami Uyar, Hatice Eyiol, Ahmet Yılmaz, Azmi Eyiol, Yakup Alsancak

**Affiliations:** 1 Department of Anaesthesiology and Reanimation, Beyhekim Training and Research Hospital, Konya 42130, Turkey; 2 Department of Cardiology, Faculty of Medicine, Karamanoğlu Mehmetbey University, Karaman 70100, Turkey; 3 Department of Cardiology, Beyhekim Training and Research Hospital, Konya 42130, Turkey; 4 Department of Cardiology, Necmettin Erbakan University, Konya 42140, Turkey

**Keywords:** sepsis, HALP score, pan-immune-inflammation value, Naples Prognostic Score, prognosis, mortality, intensive care unit, Sepsis-3

## Abstract

**Background:** Sepsis is a life-threatening organ dysfunction caused by a dysregulated host response to infection (Sepsis-3 definition), associated with high mortality in intensive care unit (ICU) patients. Composite immune–nutritional indices derived from routine laboratory data have emerged as accessible prognostic tools; however, their comparative value in critically ill septic patients remains insufficiently characterised. This study aimed to compare the prognostic performance of the haemoglobin–albumin–lymphocyte–platelet (HALP) score, pan-immune-inflammation value (PIV), and Naples Prognostic Score (NPS) for predicting in-hospital mortality in ICU patients with sepsis as the primary outcome, and to assess their incremental predictive value as the secondary objective. **Methods:** In this retrospective, two-centre cohort study, 1020 consecutive eligible adult patients fulfilling Sepsis-3 criteria (suspected or confirmed infection with an acute increase in SOFA score ≥ 2 points) admitted to the ICUs of Necmettin Erbakan University Hospital and Beyhekim Training and Research Hospital between January 2016 and June 2025 were included. HALP was calculated as haemoglobin (g/L) × albumin (g/L) × lymphocyte count (×10^9^/L) ÷ platelet count (×10^9^/L); PIV as (neutrophil × platelet × monocyte) ÷ lymphocyte (all ×10^9^/L). NPS was computed from serum albumin, neutrophil-to-lymphocyte ratio, and lymphocyte-to-monocyte ratio, with the total-cholesterol component imputed due to availability in only 31.7% of patients. Discriminative performance was evaluated by receiver operating characteristic (ROC) analysis, pairwise DeLong tests, bootstrap resampling (1000 iterations), Hosmer–Lemeshow calibration, and net reclassification improvement (NRI)/integrated discrimination improvement (IDI) analyses. Five pre-specified nested multivariable logistic regression models were constructed. **Results:** Of 1020 patients (median age 76 years, IQR 67–83; 59.8% male), 521 (51.1%) died during hospitalisation. HALP showed the highest discriminative ability among individual indices (AUC 0.626, 95% CI 0.594–0.658), while PIV was non-discriminatory (AUC 0.504, *p* = 0.78) and NPS showed limited performance (AUC 0.563, 95% CI 0.531–0.595). HALP remained an independent predictor of mortality after multivariable adjustment (OR 0.98, 95% CI 0.97–0.99, *p* = 0.002). NRI and IDI analyses showed no incremental value with NPS addition. **Conclusions:** HALP demonstrated modest but independently consistent discrimination for in-hospital mortality in ICU patients with sepsis, outperforming PIV and NPS. However, an AUC of 0.626 does not support standalone clinical use; external validation and comparison with established severity models are required before integration into risk stratification frameworks.

## 1. Introduction

Sepsis, defined by the Sepsis-3 consensus as life-threatening organ dysfunction caused by a dysregulated host response to infection, remains a leading cause of ICU mortality worldwide despite advances in supportive care [1,2]. Early and accurate risk stratification is central to sepsis management, yet established scoring systems such as APACHE II, SOFA, SAPS II, and qSOFA—while widely validated—are resource-intensive and may not capture all dimensions of immune–nutritional dysregulation that influence prognosis [1,3,4].

Several composite inflammatory–nutritional indices derived from routine complete blood count and biochemistry data have been proposed as simple, inexpensive adjuncts for prognostic assessment in critically ill patients. The HALP score integrates haemoglobin, albumin, lymphocyte count, and platelet count, simultaneously reflecting oxygen-carrying capacity, nutritional reserve, immune competence, and coagulo-inflammatory burden, and has demonstrated associations with mortality in septic patients [5,6,7]. The pan-immune-inflammation value (PIV), comprising neutrophil, platelet, monocyte, and lymphocyte counts, quantifies systemic inflammatory burden and has been evaluated in ICU patients with sepsis and sepsis-associated complications [8,9,10,11,12]. The Naples Prognostic Score (NPS), originally proposed by Galizia et al. for risk stratification in patients undergoing surgery for colorectal cancer [13], integrates serum albumin, total cholesterol, neutrophil-to-lymphocyte ratio (NLR), and lymphocyte-to-monocyte ratio (LMR); it has since been applied to cardiovascular and critical illness contexts [14,15,16,17,18,19].

Despite growing literature on each index individually, direct head-to-head comparisons of HALP, PIV, and NPS in large, real-world ICU sepsis cohorts are lacking. Understanding which index—or combination—provides the most consistent and independent discriminative value for mortality in sepsis would inform evidence-based selection of prognostic biomarkers and support parsimonious model development. Furthermore, evaluating whether any of these indices adds incremental value beyond established clinical severity scores addresses a specific clinical question not previously answered in this population.

Therefore, the primary objective of this study was to compare the discriminative performance of HALP, PIV, and NPS for predicting in-hospital mortality in critically ill patients with sepsis. The secondary objective was to assess their independent and incremental predictive value in multivariable models that include established clinical severity parameters.

## 2. Methods

### 2.1. Study Design and Setting

This was a retrospective, two-centre cohort study conducted at the ICUs of Necmettin Erbakan University Faculty of Medicine (Konya, Turkey) and Beyhekim Training and Research Hospital (Konya, Turkey). Consecutive eligible adult patients admitted between 1 January 2016 and 1 June 2025 were retrospectively identified using institutional electronic medical record systems. Both centres were covered by a single ethics approval. Data collection was retrospective and performed after ethics approval (see Section 2.6).

### 2.2. Study Population

Patients aged ≥18 years fulfilling Sepsis-3 criteria—defined as suspected or confirmed infection plus an acute increase in total SOFA score of ≥2 points from baseline—were eligible for inclusion [2]. Septic shock was defined as sepsis requiring vasopressor therapy to maintain a mean arterial pressure ≥ 65 mmHg and a serum lactate level > 2 mmol/L despite adequate fluid resuscitation. Only the first ICU admission per patient during the study period was included.

Exclusion criteria were age < 18 years; missing key admission laboratory data (complete blood count, albumin); active haematological malignancy; severe chronic inflammatory or autoimmune disease; immunosuppressive therapy at admission; incomplete clinical records; or transfer from another centre without baseline laboratory measurements.

### 2.3. Data Collection

Demographic data (age, sex), comorbidities, source of infection, microbiological culture results, and clinical severity scores (APACHE II, SOFA, SAPS II, qSOFA) were extracted from electronic records. Organ support data—including vasopressor use, mechanical ventilation, and renal replacement therapy (RRT)—and ICU and in-hospital length of stay (LOS) were obtained. All laboratory parameters were collected from samples drawn within the first 24 h of ICU admission and processed using standardised methods in each participating centre.

### 2.4. Score Calculation

Composite scores were calculated from admission laboratory values as follows.

HALP score = Haemoglobin (g/L) × Albumin (g/L) × Lymphocyte count (×10^9^/L) ÷ Platelet count (×10^9^/L). Because haemoglobin and albumin are reported in g/dL in Table 1, they were converted to g/L by multiplying by 10 before applying the formula. A lower HALP score indicates higher mortality risk.

PIV = Neutrophil count (×10^9^/L) × Platelet count (×10^9^/L) × Monocyte count (×10^9^/L) ÷ Lymphocyte count (×10^9^/L). PIV is highly right-skewed; log_10_-transformed PIV was used in regression modelling, with raw PIV reported in descriptive statistics.

Naples Prognostic Score (NPS) was originally developed for colorectal cancer patients by Galizia et al. [13] and incorporates four components, each scored 0 or 1: serum albumin (≥4.0 vs. <4.0 g/dL), total cholesterol (≥180 vs. <180 mg/dL), NLR (<3.44 vs. ≥3.44), and LMR (≥3.44 vs. <3.44). The total NPS (0–4) is treated as an ordinal variable, with higher scores indicating worse nutritional–inflammatory status and higher mortality risk, consistent with Galizia et al.’s original categorisation in which each unfavourable component (low albumin, low cholesterol, high NLR, low LMR) contributes one point.

Total cholesterol was not systematically measured at ICU admission in the participating centres, with missing data in 697 patients (68.3%). For the primary NPS analysis, the cholesterol component was assigned a score of 1 (favourable category) for all patients with missing data—an assumption that biases toward underestimating NPS severity. A pre-specified sensitivity analysis was conducted in the 323 patients (31.7%) with available cholesterol data; a complete-case analysis was also performed. Results of the sensitivity analysis are reported in Supplementary Table S1.

### 2.5. Outcome

The primary outcome was all-cause in-hospital mortality, defined as death occurring at any time during the index hospitalisation, regardless of cause. Patients were classified as survivors or non-survivors based on this endpoint.

### 2.6. Ethics

The study was approved by the Necmettin Erbakan University Non-Drug and Non-Medical Device Research Ethics Committee (approval number: 2025/6132; date: 28 November 2025). Both participating centres were included under this approval. Data were collected retrospectively using existing medical records; informed consent was waived in accordance with institutional policy. All procedures complied with the Declaration of Helsinki.

### 2.7. Statistical Analysis

Statistical analyses were performed using SPSS version 26.0 (IBM Corp., Armonk, NY, USA), MedCalc Statistical Software (Version 23.6.1), and R (version 4.3, R Foundation for Statistical Computing). Normality was assessed with the Kolmogorov–Smirnov test. Continuous variables are presented as median (interquartile range, IQR) and compared with the Mann–Whitney U test; categorical variables are presented as frequency (percentage) and compared with the chi-squared or Fisher exact test.

Multivariable logistic regression was conducted using five pre-specified nested models: Model 1 (base clinical model: age, APACHE II score, creatinine, qSOFA); Model 2 (Model 1 + HALP); Model 3 (Model 1 + log_10_-PIV); Model 4 (Model 1 + NPS); and Model 5 (Model 1 + HALP + log_10_-PIV + NPS). SOFA was excluded from all models due to collinearity with APACHE II; SAPS II was excluded due to collinearity with both APACHE II and SOFA. Because HALP is a nonlinear composite of haemoglobin, albumin, lymphocyte count, and platelet count, these individual components were not entered as separate covariates in models containing HALP, thereby avoiding overadjustment. Multicollinearity was assessed using variance inflation factors (VIF); all included variables demonstrated VIF < 5.

Discriminative performance was evaluated by ROC analysis with AUC calculation; optimal cut-offs were identified using the Youden index. Pairwise AUC comparisons were performed with the DeLong test. Model calibration was assessed using the Hosmer–Lemeshow goodness-of-fit test and calibration plots (Supplementary Figure S1). Bootstrap resampling (1000 iterations) was applied to derive optimism-corrected AUCs for all models. Incremental predictive value of NPS over the base clinical model with HALP was assessed using NRI and IDI.

All reported analyses follow TRIPOD (Transparent Reporting of a multivariable prediction model for Individual Prognosis or Diagnosis) guidelines. A two-tailed *p* < 0.05 was considered statistically significant.

## 3. Results

### 3.1. Patient Flow and Baseline Characteristics

During the study period (January 2016 to June 2025), 1248 patients with a diagnosis of sepsis were screened. After exclusion of 228 patients (active haematological malignancy, n = 54; missing key laboratory data, n = 89; immunosuppressive therapy, n = 41; transfer without baseline data, n = 44), 1020 patients were included in the final analysis (Centre 1: n = 562; Centre 2: n = 458). Of these, 521 (51.1%) died during hospitalisation and 499 (48.9%) survived. The median cohort age was 76 years (IQR 67–83); 610 patients (59.8%) were male.

The most common infection sources were respiratory (38.6%), urinary (22.4%), abdominal (18.2%), and other or unknown (20.8%); each patient was assigned a single primary source. Septic shock was present in 389 patients (38.1%). Vasopressor use, mechanical ventilation, and RRT were documented in 389 (38.1%), 512 (50.2%), and 187 (18.3%) patients, respectively. Median ICU and hospital LOS were 8 (IQR 4–16) and 14 (IQR 7–26) days, respectively.

Non-survivors were significantly older (78 vs. 72 years, *p* < 0.001) and had higher disease severity (APACHE II, SOFA, SAPS II, qSOFA; all *p* < 0.001). Haemoglobin, albumin, lymphocyte count, and platelet count were significantly lower in non-survivors, while neutrophil counts and creatinine were higher. Organ support variables and LOS are presented in Table 1; survivor vs. non-survivor comparisons for these variables were not available in the current dataset and are noted as a limitation. Full baseline characteristics are presented in Table 1.

### 3.2. Composite Score Values by Outcome Group

HALP was significantly lower in non-survivors compared with survivors (median 15 [IQR 8–26] vs. 30 [IQR 18–48]; *p* < 0.001). PIV did not differ significantly between groups (median 1900 [IQR 950–3800] vs. 1800 [IQR 850–3400]; *p* = 0.41). NPS showed modest group separation (median 3 [IQR 2–4] vs. 2 [IQR 1–2]; *p* < 0.05). Inter-index correlation analyses revealed moderate associations, indicating partial but not complete overlap in the biological domains captured.

### 3.3. Logistic Regression Analysis

In univariate analysis, age, APACHE II score, SOFA score, haemoglobin, albumin, lymphocyte count, platelet count, creatinine, CRP, qSOFA, and HALP score were significantly associated with in-hospital mortality (all *p* < 0.05). Log_10_-PIV was not significantly associated with mortality (*p* = 0.42), and NPS showed a weak univariate association (*p* = 0.02).

In the pre-specified multivariable Model 2 (base clinical model plus HALP), HALP remained an independent predictor of mortality after adjustment for age, APACHE II score, creatinine, and qSOFA (OR 0.98, 95% CI 0.97–0.99, *p* = 0.002). Neither log_10_-PIV in Model 3 nor NPS in Model 4 reached statistical significance. In the fully combined Model 5, only HALP and age remained significant; log_10_-PIV and NPS remained non-significant. Full multivariable results are presented in Table 2.

Because HALP is a nonlinear composite of haemoglobin, albumin, lymphocytes, and platelets, these individual components were excluded from models containing HALP to avoid overadjustment. The independent effect of HALP therefore reflects the value of the composite index as a whole.

### 3.4. ROC Curve Analysis and Discrimination

**Figure 1:**
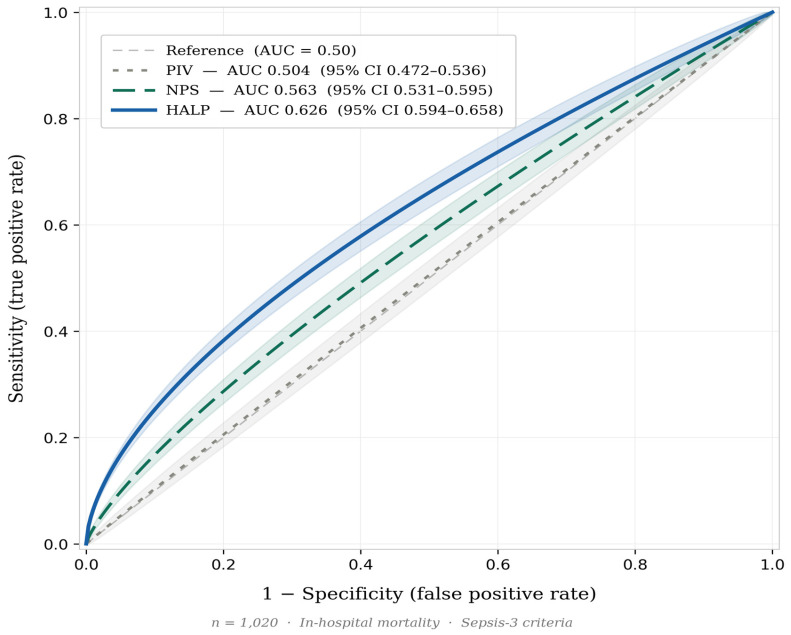
Receiver operating characteristic (ROC) curves for HALP score, PIV, and Naples Prognostic Score (NPS) for predicting in-hospital mortality in ICU patients with Sepsis-3 defined sepsis (n = 1020). AUCs with 95% confidence bands are displayed for each individual index. HALP (AUC 0.626) demonstrated the highest discriminative performance; PIV (AUC 0.504) was non-discriminatory. The diagonal dashed line represents chance-level discrimination (AUC = 0.50). A lower HALP score indicates higher mortality risk. Calibration plots for all models are provided as Supplementary Figure S1.

Among individual indices, HALP demonstrated the highest discriminative ability (AUC 0.626, 95% CI 0.594–0.658), reflecting modest but statistically significant discrimination. PIV showed performance no better than chance (AUC 0.504, 95% CI 0.472–0.536; *p* = 0.78). NPS exhibited limited discrimination (AUC 0.563, 95% CI 0.531–0.595). Bootstrap optimism-corrected AUCs were 0.619 (95% CI 0.588–0.650) for HALP, 0.498 for PIV, and 0.557 for NPS, consistent with the primary estimates. Pairwise DeLong comparisons are presented in Table 3. Hosmer–Lemeshow testing indicated adequate calibration for all HALP-containing models (*p* > 0.05); calibration plots are provided as Figure 1.

### 3.5. Incremental Value Analysis

Neither NRI nor IDI analyses demonstrated statistically significant improvement in risk classification upon addition of NPS to the base clinical model containing HALP (Model 2 → Model 5: NRI overall +0.021, 95% CI −0.034 to +0.076, *p* = 0.46; IDI +0.003, 95% CI −0.002 to +0.008, *p* = 0.28), confirming that NPS does not provide meaningful incremental value in this cohort (Table 4 and Table 5).

## 4. Discussion

In this retrospective two-centre cohort study of 1020 ICU patients with Sepsis-3 defined sepsis, the principal findings are threefold. First, HALP demonstrated the most consistent independent prognostic signal, with an AUC of 0.626 and independent retention across pre-specified multivariable models—though this level of discrimination is modest and insufficient, by itself, to justify clinical implementation without external validation. Second, PIV showed no meaningful discriminative performance, and the addition of log-PIV did not improve any model. Third, NPS did not provide incremental predictive value beyond HALP in either NRI or IDI analyses.

The modest but consistent performance of HALP likely reflects its multidimensional biological composition: haemoglobin captures oxygen delivery capacity, albumin reflects nutritional status and hepatic synthetic function, lymphocyte count represents adaptive immune competence, and platelet count integrates coagulo-inflammatory responses. This simultaneous integration of multiple pathophysiological domains relevant to sepsis may enable HALP to capture prognostic information that single-domain markers cannot. The present findings are consistent with several recent studies demonstrating a significant association between HALP score and mortality in septic patients [5,6,7].

Beyond sepsis, the prognostic relevance of HALP has been demonstrated in oncological settings including pancreatic and colorectal cancer [20,21,22,23], further supporting the biological plausibility of HALP as a multidimensional prognostic marker reflecting inflammation, immune competence, and nutritional reserve across different clinical contexts.

The non-discriminatory performance of PIV (AUC ≈ 0.50) in this cohort contrasts with some previously reported positive associations in ICU patients with septic shock and sepsis-associated acute kidney injury [8,9,10,11,12]. This discrepancy may reflect the demographic profile of the cohort (elderly patients, high disease severity, 51.1% mortality) or the high degree of variability in inflammatory markers within a heterogeneous ICU population. The near-random discriminative performance suggests that, in this setting, PIV may reflect generalised inflammatory activation rather than mortality-specific biology.

The NPS, originally developed and validated in colorectal cancer patients by Galizia et al. [13], showed limited performance in this critically ill sepsis cohort. Its prognostic value appears context-dependent. Importantly, in this critically ill cohort, higher NPS values were associated with greater mortality risk (OR 1.15, *p* = 0.02; non-survivors median NPS 3 vs. survivors median 2), consistent with the original Galizia et al. coding in which each unfavourable component (albumin below 4.0 g/dL, cholesterol below 180 mg/dL, NLR ≥ 3.44, LMR below 3.44) contributes one point; the near-universal presence of hypoalbuminaemia and elevated NLR in ICU patients means the score is largely driven by these unfavourable components, which explains the observed association with mortality. The nutritional and inflammatory components of NPS are largely captured by HALP in this population. A major methodological limitation was the absence of systematic total cholesterol measurement at ICU admission, available in only 31.7% of patients, necessitating an imputation strategy for the primary analysis. The sensitivity analysis in complete-case patients is therefore critical for assessing the validity of the primary NPS results; these analyses are presented in Supplementary Table S1. NPS has shown prognostic associations in cardiovascular and neurological settings [14,15,16,17,18,19,24], and its exploratory application here in an ICU sepsis cohort should be interpreted in that context.

From a clinical perspective, these findings support HALP as a simple, low-cost adjunct marker for mortality risk in ICU patients with sepsis. However, an AUC of 0.626 indicates limited standalone discrimination; HALP should be considered a complement to—rather than a replacement for—established severity scores such as APACHE II and SOFA. Future studies should evaluate whether HALP improves calibration or reclassification when added to a full clinical severity model using decision curve analysis and should perform external validation in independent cohorts before recommending clinical adoption.

### Limitations

Several limitations should be acknowledged. First, the retrospective design is susceptible to selection and information bias. Second, total cholesterol was not systematically available, necessitating an imputation strategy for the primary NPS analysis; the cholesterol-related NPS component results should be interpreted cautiously. Third, survivor vs. non-survivor comparisons for organ support variables and LOS were not available in the current dataset. Fourth, all biomarkers were assessed at a single time point; temporal dynamics were not evaluated. Fifth, although bootstrap internal validation was performed, external validation in an independent cohort is required to confirm generalisability. Sixth, certain variables—including body mass index, steroid exposure, and detailed microbiological characterisation—were not consistently available across both centres. Seventh, the high in-hospital mortality (51.1%) in this elderly cohort (median age 76 years) may limit external validity to younger or less severely ill populations.

## 5. Conclusions

The HALP score demonstrated modest but independent discrimination for in-hospital mortality in critically ill patients with Sepsis-3 defined sepsis, outperforming PIV and the Naples Prognostic Score. An AUC of 0.626 does not support clinical use of HALP as a standalone risk stratification tool; external validation and comparison with full clinical severity models are required. PIV showed no meaningful prognostic utility in this cohort, and its addition to models did not improve performance. NPS did not provide incremental value beyond HALP. These findings underscore the importance of rigorous, pre-specified model construction and transparent reporting in prognostic biomarker research in critical care.

## Figures and Tables

**Table 1:** Baseline Characteristics of the Study Population According to In-Hospital Survival Status.

Variable	Overall (n = 1020)	Survivors (n = 499)	Non-Survivors (n = 521)	*p*-Value
**Demographics**				
Age, years	76 (67–83)	72 (64–80)	78 (70–85)	<0.001
Male sex, n (%)	610 (59.8)	290 (58.1)	320 (61.4)	0.28
**Clinical Severity**				
APACHE II score	38 (30–46)	32 (26–40)	42 (35–50)	<0.001
SOFA score	9 (6–12)	7 (5–10)	11 (8–14)	<0.001
SAPS II score	48 (35–68)	35 (30–37)	68 (55–78)	<0.001
qSOFA score	2 (1–3)	1 (1–1)	3 (2–3)	<0.001
Septic shock, n (%)	389 (38.1)	128 (25.7)	261 (50.1)	<0.001
**Organ Support ^a^**				
Vasopressor use, n (%)	389 (38.1)	N/A	N/A	N/A
Mechanical ventilation, n (%)	512 (50.2)	N/A	N/A	N/A
RRT, n (%)	187 (18.3)	N/A	N/A	N/A
ICU LOS, days	8 (4–16)	N/A	N/A	N/A
Hospital LOS, days	14 (7–26)	N/A	N/A	N/A
**Haematological Parameters**				
Haemoglobin, g/dL	10.9 (9.4–12.4)	11.5 (10.0–12.9)	10.3 (8.9–11.8)	<0.001
Neutrophil, ×10^9^/L	9.8 (6.5–14.2)	8.9 (6.0–12.5)	10.7 (7.2–15.6)	<0.001
Lymphocyte, ×10^9^/L	0.9 (0.6–1.4)	1.1 (0.7–1.6)	0.8 (0.5–1.2)	<0.001
Monocyte, ×10^9^/L	0.5 (0.3–0.7)	0.5 (0.3–0.7)	0.5 (0.3–0.8)	0.09
Platelet, ×10^9^/L	190 (130–260)	210 (150–280)	170 (110–230)	<0.001
**Biochemical Parameters**				
Albumin, g/dL	2.9 (2.4–3.3)	3.1 (2.6–3.5)	2.7 (2.2–3.1)	<0.001
Creatinine, mg/dL	1.3 (0.9–2.1)	1.2 (0.8–1.9)	1.5 (1.0–2.4)	<0.001
CRP, mg/L	145 (85–210)	130 (75–195)	160 (95–230)	<0.01
**Composite Scores**				
HALP score ^b^	22 (12–38)	30 (18–48)	15 (8–26)	<0.001
PIV (raw)	1850 (900–3600)	1800 (850–3400)	1900 (950–3800)	0.41
Naples Prognostic Score ^c^	2 (1–3)	2 (1–2)	3 (2–4)	<0.05

Continuous variables: median (IQR). Categorical variables: n (%). Comparisons: Mann–Whitney U or chi-squared/Fisher exact as appropriate. ^a^ Survivor vs. non-survivor comparisons for organ support variables and LOS were not available in the current dataset; overall cohort values only are reported. ^b^ HALP = Hb (g/L) × albumin (g/L) × lymphocyte (×10^9^/L) ÷ platelet (×10^9^/L); haemoglobin and albumin as reported in g/dL are multiplied by 10 to convert to g/L before calculation. ^c^ NPS is an ordinal variable (0–4); higher scores indicate better nutritional–inflammatory status. APACHE II, Acute Physiology and Chronic Health Evaluation II; CRP, C-reactive protein; HALP, haemoglobin–albumin–lymphocyte–platelet; ICU, intensive care unit; IQR, interquartile range; LOS, length of stay; NPS, Naples Prognostic Score; PIV, pan-immune-inflammation value; RRT, renal replacement therapy; SOFA, Sequential Organ Failure Assessment; qSOFA, Quick SOFA; SAPS II, Simplified Acute Physiology Score II. N/A, not applicable.

**Table 2:** Multivariable Logistic Regression Analysis for In-Hospital Mortality: Pre-specified Models.

Variable	Univariate OR (95% CI)	*p*	Model 2: Base + HALP OR (95% CI)	*p*	Model 3: Base + log-PIV OR (95% CI)	*p*	Model 4: Base + NPS OR (95% CI)	*p*
Age, per year	1.045 (1.032–1.058)	<0.001	1.032 (1.018–1.046)	<0.001	1.041 (1.027–1.055)	<0.001	1.038 (1.024–1.052)	<0.001
APACHE II score	1.08 (1.06–1.10)	<0.001	1.06 (1.04–1.08)	<0.001	1.07 (1.05–1.09)	<0.001	1.07 (1.05–1.09)	<0.001
Creatinine, mg/dL	1.21 (1.12–1.30)	<0.001	1.10 (1.02–1.19)	0.01	1.12 (1.03–1.21)	0.007	1.11 (1.03–1.20)	0.009
qSOFA score	1.85 (1.60–2.10)	<0.001	1.32 (1.10–1.58)	0.002	1.44 (1.21–1.71)	<0.001	1.40 (1.18–1.67)	<0.001
HALP score	0.97 (0.96–0.98)	<0.001	0.98 (0.97–0.99)	0.002	—	—	—	—
log_10_-PIV	1.09 (0.88–1.35)	0.42	—	—	1.11 (0.90–1.38)	0.33	—	—
NPS (ordinal, 0–4)	1.15 (1.02–1.29)	0.02	—	—	—	—	1.05 (0.92–1.20)	0.46

Model 1 (base clinical model): age, APACHE II score, creatinine, qSOFA. All models use Model 1 as base. Model 5 (all indices combined) is presented in Supplementary Table S2. SOFA excluded due to collinearity with APACHE II; SAPS II excluded due to collinearity with both. HALP component variables (haemoglobin, albumin, lymphocyte, platelet) not entered in models containing HALP to avoid overadjustment. All VIF < 5. NPS, Naples Prognostic Score; OR, odds ratio; CI, confidence interval; VIF, variance inflation factor.

**Table 3:** Pairwise AUC Comparisons (DeLong Test).

Comparison	ΔAUC	*p*-Value
HALP vs. PIV	+0.122	<0.001
HALP vs. NPS	+0.063	0.02
PIV vs. NPS	−0.059	0.04

NPS, Naples Prognostic Score; HALP, haemoglobin–albumin–lymphocyte–platelet score; PIV, pan-immune-inflammation value.

**Table 4:** Incremental Prognostic Value of NPS Beyond the Base Clinical Model with HALP (Model 2 → Model 5).

Metric	Model Comparison	Estimate	95% CI	*p*-Value
NRI (overall)	Model 2 (base + HALP) → Model 5 (base + HALP + log-PIV + NPS)	+0.021	−0.034 to +0.076	0.46
NRI (events)	Death reclassification, Model 2 → Model 5	+0.015	−0.028 to +0.058	0.49
NRI (non-events)	Survivor reclassification, Model 2 → Model 5	+0.006	−0.021 to +0.033	0.62
IDI	Model 2 (base + HALP) → Model 5 (base + HALP + log-PIV + NPS)	+0.003	−0.002 to +0.008	0.28

NRI, net reclassification improvement; IDI, integrated discrimination improvement; CI, confidence interval; NPS, Naples Prognostic Score; HALP, haemoglobin–albumin–lymphocyte–platelet score. Neither NRI nor IDI reached statistical significance, confirming that the addition of log-PIV and NPS to a model containing HALP does not improve risk classification.

**Table 5:** ROC Curve Analysis: Discriminative Performance for In-Hospital Mortality.

Index/Model	AUC (95% CI)	Cut-Off	Sensitivity (%)	Specificity (%)	*p* (vs. 0.50)
HALP	0.626 (0.594–0.658)	24.5	68	55	<0.001
HALP (bootstrap-corrected)	0.619 (0.588–0.650)	—	—	—	<0.001
PIV (raw)	0.504 (0.472–0.536)	N/A	N/A	N/A	0.78
PIV (bootstrap-corrected)	0.498 (0.467–0.530)	—	—	—	0.91
Naples Prognostic Score	0.563 (0.531–0.595)	≥3	60	52	0.01
NPS (bootstrap-corrected)	0.557 (0.525–0.589)	—	—	—	0.02

AUC, area under the curve; CI, confidence interval; N/A, not applicable (AUC not significantly different from 0.50). Bootstrap optimism correction based on 1000 resamples. Optimal cut-offs determined by Youden index. NPS, Naples Prognostic Score; PIV, pan-immune-inflammation value.

## Data Availability

The datasets analysed during this study are available from the corresponding author on reasonable request.

## Supplementary Materials

The following supporting information can be downloaded at: https://www.mdpi.com/article/10.3390/jcm15124729/s1, Figure S1. Calibration Plots for Logistic Regression Models Predicting In-Hospital Mortality; Table S1. Sensitivity Analysis of the Naples Prognostic Score (NPS) in Patients with Available Total Cholesterol Data (n = 323) versus Primary Imputed Analysis (n = 1020); Table S2. Full Multivariable Logistic Regression: Model 5 (Base Clinical Model + HALP + log_10_-PIV + NPS).

## Abbreviations

APACHE II, Acute Physiology and Chronic Health Evaluation II; AUC, area under the curve; CI, confidence interval; CRP, C-reactive protein; HALP, haemoglobin–albumin–lymphocyte–platelet score; ICU, intensive care unit; IDI, integrated discrimination improvement; IQR, interquartile range; LMR, lymphocyte-to-monocyte ratio; LOS, length of stay; NLR, neutrophil-to-lymphocyte ratio; NPS, Naples Prognostic Score; NRI, net reclassification improvement; OR, odds ratio; PIV, pan-immune-inflammation value; ROC, receiver operating characteristic; RRT, renal replacement therapy; SOFA, Sequential Organ Failure Assessment; VIF, variance inflation factor.
