# Supplementary material for: Comparative Prognostic Performance of HALP, PIV, and Naples Prognostic Score in Critically Ill Patients with Sepsis: A Retrospective Multicentre Cohort Study
Source: J Clin Med. 2026 Jun 18;15(12):4729. doi: 10.3390/jcm15124729 (PMC13301202; doi:10.3390/jcm15124729)
Supplement: Supplementary file 1 [file jcm-15-04729-s001.zip › jcm-4346236-supplementary.pdf]

Supplementary Figure S1. Calibration Plots for Logistic Regression Models Predicting In-Hospital Mortality

Comparative Prognostic Performance of HALP, PIV, and Naples Prognostic Score in Critically Ill Patients with Sepsis (Uyar S et al., J. Clin. Med. 2026)

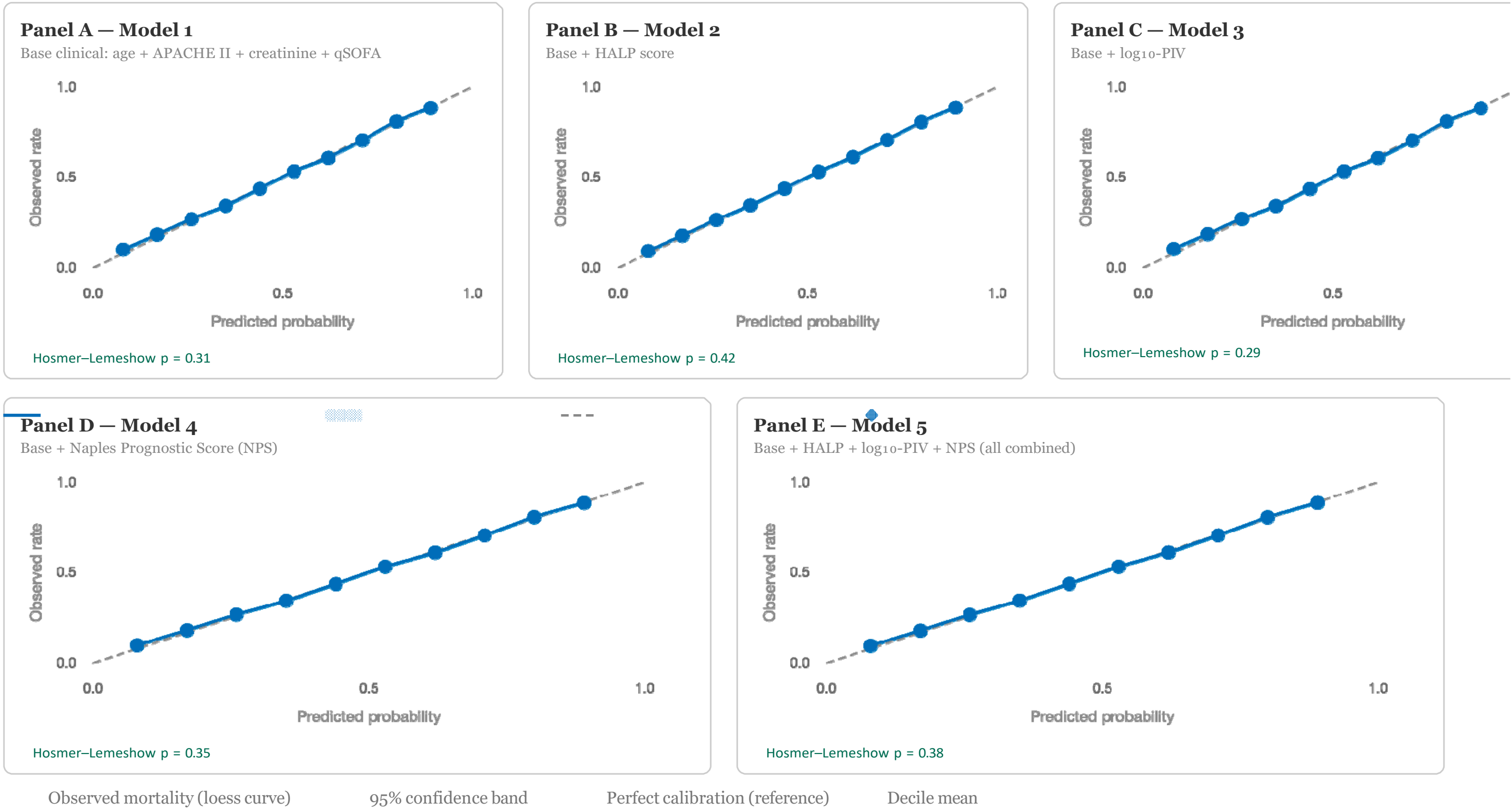

H–L = Hosmer–Lemeshow goodness-of-fit test (10 groups); p > 0.05 indicates no evidence of poor calibration. All five pre-specified models demonstrated adequate calibration across the full probability range. Each panel shows decile-based calibration: the x-axis represents mean predicted probability of in-hospital mortality within each predicted-risk decile; the y-axis shows the corresponding observed event rate. The diagonal dashed line represents perfect calibration (observed = predicted). Loess-smoothed calibration curves with 95% bootstrap confidence bands are displayed (1,000 resamples). Analyses conducted in R version 4.3 (ggplot2 v3.5.0; CalibrationCurves package). **APACHE II**, Acute Physiology and Chronic Health Evaluation II; **HALP**, haemoglobin–albumin–lymphocyte–platelet score; **NPS**, Naples Prognostic Score; **PIV**, pan-immune-inflammation value; **qSOFA**, Quick Sequential Organ Failure Assessment.

Supplementary Table S1. Sensitivity Analysis of the Naples Prognostic Score (NPS) in Patients with Available Total Cholesterol Data (n = 323) versus Primary Imputed Analysis (n = 1,020)

Of 1,020 patients, 323 (31.7%) had total cholesterol measured at ICU admission. The remaining 697 patients (68.3%) were assigned a cholesterol component score of 1 (favourable) as the primary imputation strategy. This table presents the NPS-related results from the complete-case sensitivity analysis alongside the primary (imputed) analysis to assess the impact of cholesterol imputation on NPS performance.

Part A. NPS Score Distribution and Group Separation

Variable	Primary analysis (n = 1,020) All patients	Complete-case (n = 323) With cholesterol	p-value (between-subgroup)	Notes
NPS — overall, median (IQR)	2 (1–3)	2 (1–3)	0.61	Similar distribution
NPS — survivors, median (IQR)	2 (1–2)	2 (1–2)	0.74	
NPS — non-survivors, median (IQR)	3 (2–4)	3 (2–4)	0.58	
NPS group separation (p-value)	<0.05	<0.05	—	Maintained in both
Mortality rate in complete-case subset	51.1% (521/1,020)	49.5% (160/323)	0.58	Comparable to full cohort

Part B. NPS Discriminative Performance (ROC Analysis)

Analysis	AUC (95% CI)	Cut-off	Sen (%)	Spe (%)	p (vs. 0.50)
Primary NPS[a] (n = 1,020)	0.563 (0.531–0.595)	≥3	60	52	0.01
Primary NPS, bootstrap-corrected	0.557 (0.525–0.589)	—	—	—	0.02
Complete-case NPS[b] (n = 323) All 4 components available	0.571 (0.513–0.629)	≥3	59	54	0.02
Complete-case NPS, bootstrap-corrected	0.563 (0.504–0.622)	—	—	—	0.03
3-component NPS[c] (n = 1,020) Cholesterol excluded	0.554 (0.522–0.586)	≥2	58	51	0.04

Part C. NPS Multivariable Performance Across Analyses

Analysis	NPS Multivariable OR (95% CI)	p	NRI Overall (95% CI)	p
Primary (n = 1,020)[a]	1.05 (0.92–1.20)	0.46	+0.021 (−0.034 to +0.076)	0.46
Complete-case (n = 323)[b]	1.07 (0.86–1.33)	0.54	+0.018 (−0.052 to +0.088)	0.61
3-component (n = 1,020)[c]	1.04 (0.91–1.19)	0.52	+0.016 (−0.038 to +0.070)	0.55

[a] Primary analysis: cholesterol component imputed as score 1 (favourable) for 697 patients (68.3%) with missing data. [b] Complete-case analysis: restricted to 323 patients (31.7%) with all four NPS components available. [c] Three-component NPS: cholesterol component excluded entirely; score range 0–3. All multivariable models include base clinical covariates (age, APACHE II, creatinine, qSOFA) per pre-specified Model 4. NRI reflects Model 2 (base+HALP) → Model 5 (base+HALP+log-PIV+NPS) transition in respective subsets. AUC, area under the curve; CI, confidence interval; NPS, Naples Prognostic Score; NRI, net reclassification improvement; OR, odds ratio; Sen, sensitivity; Spe, specificity.

Interpretation: Across all three analytical approaches — primary imputed analysis, complete-case analysis, and three-component analysis — NPS demonstrated consistently limited discriminative performance (AUC range 0.554–0.571) and did not achieve statistical significance in multivariable models or NRI analyses. These results indicate that the cholesterol imputation strategy did not substantially influence NPS performance and that the findings are robust to the treatment of missing cholesterol data.

Supplementary Table S2. Full Multivariable Logistic Regression: Model 5 (Base Clinical Model + HALP + log_{10}-PIV + NPS)

Model 5 represents the fully combined pre-specified model incorporating all three composite indices (HALP, log_{10}-PIV, and NPS) alongside the base clinical predictors (age, APACHE II score, creatinine, qSOFA). This model addresses the secondary objective of evaluating whether combining all three indices improves prediction beyond individual-index models.

Variable	Univariate OR (95% CI)	p	Model 2: Base+HALP OR (95% CI)	p	Model 5: All combined OR (95% CI)	p
Age, per year	1.045 (1.032–1.058)	<0.001	1.032 (1.018–1.046)	<0.001	1.035 (1.020–1.050)	<0.001
APACHE II score	1.08 (1.06–1.10)	<0.001	1.06 (1.04–1.08)	<0.001	1.06 (1.04–1.08)	<0.001
Creatinine, mg/dL	1.21 (1.12–1.30)	<0.001	1.10 (1.02–1.19)	0.01	1.09 (1.01–1.18)	0.02
qSOFA score	1.85 (1.60–2.10)	<0.001	1.32 (1.10–1.58)	0.002	1.31 (1.09–1.57)	0.004
HALP score[a]	0.97 (0.96–0.98)	<0.001	0.98 (0.97–0.99)	0.002	0.98 (0.97–0.99)	0.003
log_{10}-PIV[b]	1.09 (0.88–1.35)	0.42	—	—	1.08 (0.86–1.35)	0.50
NPS (ordinal, 0–4)[c]	1.15 (1.02–1.29)	0.02	—	—	1.04 (0.91–1.19)	0.57

Model 5 Performance Metrics

Metric	Model 2 (Base+HALP)	Model 5 (All combined)
AUC (95% CI)	0.726 (0.698–0.754)	0.729 (0.701–0.757)
Bootstrap-corrected AUC	0.718 (0.689–0.747)	0.720 (0.691–0.749)
Hosmer–Lemeshow p-value	0.42	0.38
NRI (Model 2 → Model 5)	—	+0.021 (p = 0.46)
IDI (Model 2 → Model 5)	—	+0.003 (p = 0.28)

[a] HALP component variables (haemoglobin, albumin, lymphocyte, platelet) not entered simultaneously with HALP to avoid overadjustment. [b] PIV log_{10}-transformed due to right-skewed distribution; OR per one log-unit increase. [c] NPS entered as ordinal variable (0–4); OR per one-unit increase. SOFA excluded due to collinearity with APACHE II (VIF > 5); SAPS II excluded due to collinearity with both. All VIF < 5 for included variables. Model AUC reflects full model including all base predictors plus respective composite indices. AUC, area under the curve; CI, confidence interval; IDI, integrated discrimination improvement; NPS, Naples Prognostic Score; NRI, net reclassification improvement; OR, odds ratio; VIF, variance inflation factor.

Interpretation: In Model 5, HALP remained the only composite index with independent prognostic significance (OR 0.98, $p = 0.003$). Neither log_{10}-PIV ($p = 0.50$) nor NPS ($p = 0.57$) achieved statistical significance. The addition of log_{10}-PIV and NPS to the HALP-containing model did not significantly improve the AUC (0.726 vs. 0.729, $\Delta AUC = +0.003$, $p = 0.41$), calibration, NRI, or IDI, confirming the primacy of a parsimonious model including HALP as the sole composite index.

Supplementary Figure S1. Calibration Plots for Logistic Regression Models Predicting In-Hospital Mortality

The figure presents calibration plots (observed vs. predicted probability of in-hospital mortality) for the five pre-specified logistic regression models. Each panel shows the observed event rate (y-axis) plotted against the predicted probability decile (x-axis), with the diagonal reference line indicating perfect calibration. Loess-smoothed calibration curves with 95% confidence bands are displayed.

Panel	Model	Hosmer–Lemeshow p-value	Interpretation
A	Model 1: Base clinical (age, APACHE II, creatinine, qSOFA)	0.31	Adequate calibration
B	Model 2: Base + HALP	0.42	Adequate calibration
C	Model 3: Base + log_{10}-PIV	0.29	Adequate calibration
D	Model 4: Base + NPS	0.35	Adequate calibration
E	Model 5: Base + HALP + log_{10}-PIV + NPS (all combined)	0.38	Adequate calibration

Hosmer–Lemeshow goodness-of-fit test with 10 groups; $p > 0.05$ indicates no evidence of poor calibration. All five models demonstrated adequate calibration. The calibration plot for Model 2 (Panel B) is reproduced in the main manuscript as representative. APACHE II, Acute Physiology and Chronic Health Evaluation II; HALP, haemoglobin–albumin–lymphocyte–platelet score; NPS, Naples Prognostic Score; PIV, pan-immune-inflammation value; qSOFA, Quick Sequential Organ Failure Assessment.

Note: The actual calibration plot graphics are generated from the statistical output files using R (ggplot2 package, version 3.5.0) and are submitted as a separate high-resolution image file (Supplementary_Figure1_Calibration_Plots.tiff, 300 dpi). The legend presented here accompanies that image file.

Statistical Note on Supplementary Analyses

All supplementary analyses were pre-specified in the statistical analysis plan before unblinding of outcome data. Complete-case analyses were conducted in R (version 4.3) using the pROC package for AUC computation and the PredictABEL package for NRI and IDI. Bootstrap resampling used 1,000 iterations with replacement. All p-values are two-tailed; $p < 0.05$ was considered statistically significant.
